# Aspirations of the ilium and proximal femur increase the likelihood of culturing an organism in patients with presumed septic arthritis of the hip

**DOI:** 10.1007/s11832-015-0669-5

**Published:** 2015-07-14

**Authors:** Gregory A. Schmale, Viviana Bompadre

**Affiliations:** Department of Orthopedics and Sports Medicine, Seattle Children’s Hospital, 4800 Sand Point Way NE, M/S OA.9.120, Seattle, WA 98105 USA; Department of Orthopedics and Sports Medicine, University of Washington, Seattle, WA USA

**Keywords:** Septic hip, Peri-articular hip infection, Bone and joint aspirations, Septic arthritis, Osteomyelitis

## Abstract

**Purpose:**

To test the hypothesis that collecting material for culture from metaphyseal bone of the ilium and proximal femur at the time of a hip aspiration will increase the sensitivity to detect an infectious organism in patients with presumed septic arthritis of the hip.

**Methods:**

We retrospectively reviewed a series of 36 patients with presumed septic arthritis of the hip, based on clinical exam and serum inflammatory markers, who underwent aspirations of hip synovial fluid as well as blood from the ilium and proximal femur. Culture results from aspirates of synovial fluid and bone and tissue from capsule were compared to determine the sensitivities and specificities of a synovial aspirate alone versus synovial aspirate plus aspirates of the ilium and proximal femur to detect infection.

**Results:**

The sensitivity of hip synovial fluid aspirates to detect infection via positive culture was only 63 %, though this increased significantly to 100 % when the results of cultures of aspirates of the ilium and proximal femur were included. The specificities were equivalent in both modalities (≥90 %). We conclude that obtaining aspirates of the ilium and proximal femur at the time of hip synovial fluid aspiration increases the likelihood that the procedure will return an infectious organism.

**Conclusion:**

Positive cultures from a child with a septic hip or peri-articular hip infection help to efficiently and effectively guide antibiotic treatment. The child with a septic hip or peri-articular hip infection and positive cultures is likely to receive more narrow-spectrum therapy, potentially decreasing the overuse of broad-spectrum antibiotics.

**Level of evidence: diagnostic study level III:**

Development of diagnostic criteria on the basis of a series of non-consecutive patients (with universally applied reference “gold standard”).

## Introduction

Differentiating between infectious and non-infectious causes of hip pain is challenging. High mortality rates in infants and children with septic arthritis of the hip were first described by Smith in the late 1800s [1]. Though mortality rates have dropped to near zero [2, 3], the potential morbidity of septic arthritis of the hip remains high [2, 4–18] and identification of the offending organism challenging [19]. Numerous studies indicate that cultures may be positive in only 30–60 % of patients [20]. Identifying the causative organism in a presumed bone and joint infection may reduce unnecessarily broad antibiotic regimens, regimens that risk the development of antibiotic resistance as well as potentially generating unnecessarily high costs of treatment.

Hip aspiration is a commonly performed procedure in the work-up of an irritable hip with an effusion. Aspirates of hip synovial fluid with high cell counts suggest a probable septic arthritis of the hip; high synovial fluid white cell counts from hip joint aspiration in concert with a positive blood culture establish a definite diagnosis of septic arthritis of the hip [21, 22]. It has been suggested that septic arthritis and peri-articular osteomyelitis may be two forms of the same disease process [23]. Accepting that this notion may be true, and recognising that, in the absence of an abscess of bone in peri-articular osteomyelitis about the hip we treat the two conditions in the same fashion after drainage of the joint, we propose that aspiration of metaphyseal bone near the hip should increase the likelihood of identifying the infectious organism for patients with a presumed septic arthritis, thereby aiding in the narrowing of antibiotic treatment, optimising care. Our hypothesis is that collecting specimens for culture of synovial fluid and blood—not only from the peripheral vasculature, but also from metaphyseal bone of the ilium and proximal femur at the time of a hip joint aspiration—will increase the sensitivity of the procedure to detect an infectious organism.

## Materials and methods

**Fig. 1:**
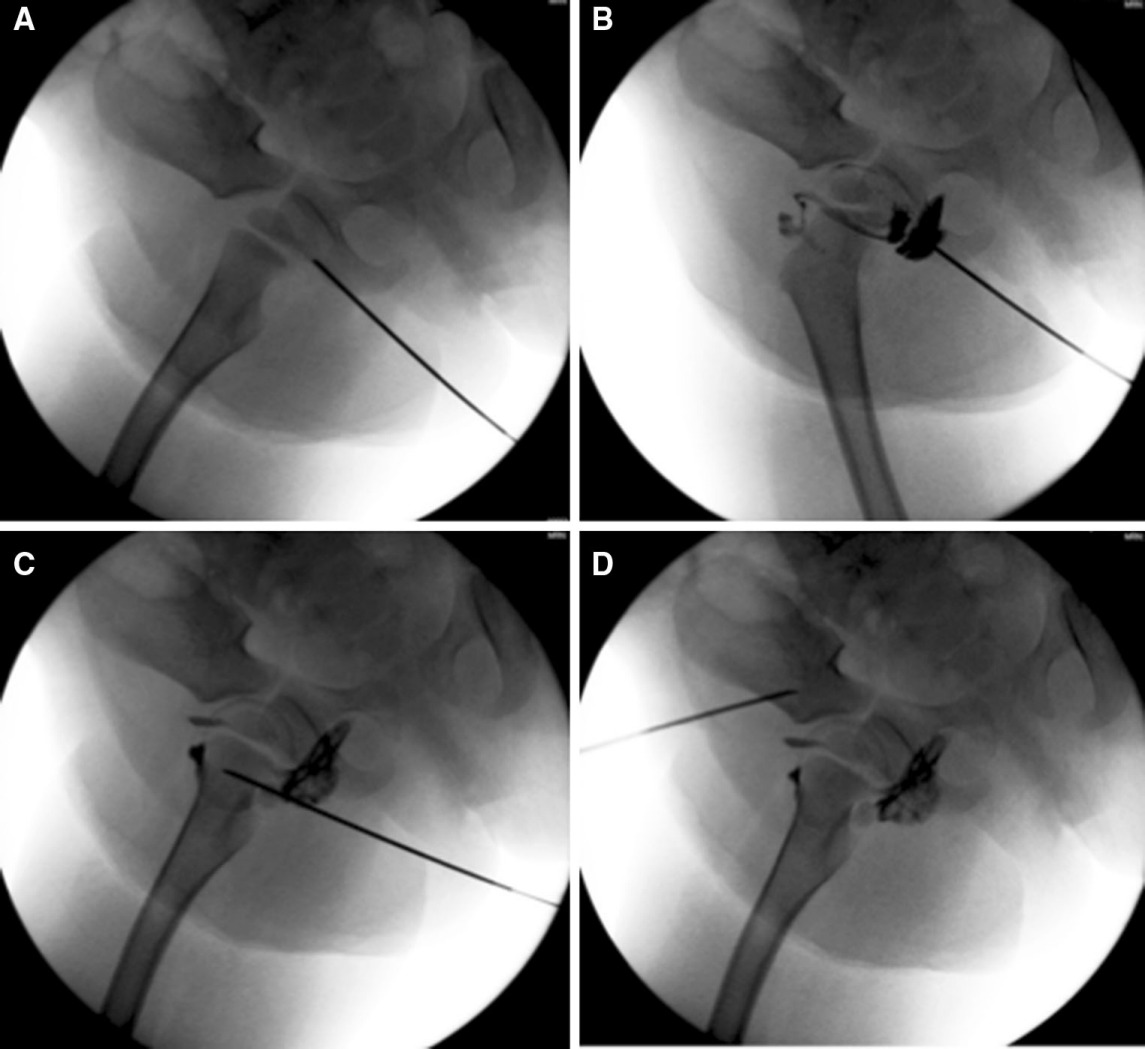
**a** Fluoroscopic image of the position of the 18-gauge spinal needle for a hip joint aspiration via a sub-adductor approach. **b** Fluoroscopic image of the hip arthrogram using a mixture of radio-opaque dye and saline. **c** Fluoroscopic image of needle position for aspiration of medullary blood from the proximal femur. **d** Fluoroscopic image of needle position for aspiration of medullary blood from the ilium

This is a retrospective review of all patients (84) treated surgically by one surgeon at one children’s hospital for a diagnosis of probable septic arthritis of the hip between 2001 and 2012. Of these 84 patients, a subset of 36 patients aged 4–21 years with 37 treated hips was identified and included as they were treated not only by hip aspiration, but aspiration of peri-articular bone (Fig. 1) and open wash-out of the hip because of purulence of the joint aspirate. All aspirates and specimens from the hip joint capsules were then sent for culture. This subset of patients with suspected septic arthritis of the hip included all those that had the most extensive culturing of tissues, i.e. including cultures from aspirates of synovial fluid, blood from the ilium to proximal femur and a hip joint capsule, so that the added value of aspirations of bone about the hip could best be determined.

Patients typically had an irritable hip and approximately three or more positive symptoms and signs of a septic hip (temperature >38.5 °C during the preceding week, refusal to ambulate at presentation, serum WBC count >12,000 cells/mm^3^, erythrocyte sedimentation rate >40 mm/h and C-reactive protein >20.0 mg/l) as described by Kocher [24] and Caird [22], though an irritable hip with an effusion also typically led to treatment. Patients were not excluded because of an underlying diagnosis such as cancer, immune deficiency, idiopathic inflammatory arthritis or recent antibiotic treatment.

The gold standard for a peri-articular hip infection was set as any one of the following: a positive culture from the hip joint capsule, joint aspirate, blood cultured from an aspiration of the ilium or proximal femur, or joint aspirate of greater than 50,000 white blood cells with a positive blood culture. Septic arthritis was defined as present if there were a positive culture from the hip joint capsule or joint fluid, or a joint aspirate of greater than 50,000 white blood cells with a positive culture from peripheral blood, the ilium or proximal femur. Positive cultures from the ilium and/or femur in the absence of a positive culture from the hip and less than 50,000 white blood cells were deemed a ‘peri-articular hip infection without septic arthritis.’ The final diagnoses were gleaned from the medical records.

Culture results from aspirates of synovial fluid and bone were compared to determine the sensitivities and specificities of a synovial aspirate alone versus synovial aspirate plus aspirates of the ilium and proximal femur to detect peri-articular hip infection. Sensitivities and specificities were calculated using 2 × 2 tables, and 95 % confidence intervals (CIs) were reported. We compared the sensitivity values of joint aspirates (Modality 1) with joint aspirates plus aspirates of the ilium and proximal femur (Modality 2) using receiver operating characteristic (ROC) curves. A Chi-square test was used to determine significant differences between the areas under the curves (AUCs). Statistical significance was set at *p* ≤ 0.05 and all confidence intervals at 95 %. All analyses were performed using STATA 12.0 (StataCorp LP, College Station, TX, USA).

### Surgical technique

Aspirations were performed by the primary author in the operating room under general anaesthesia with fluoroscopic guidance using primarily 18-gauge spinal needles. Separate needles were used for each aspiration. Joint aspirates were accompanied by injection of arthrogram dye only if there were uncertainty regarding the position of the needle tip. No attempts at performing extra-capsular aspirations of bone were made. Spinal needles with the introducer or obturator in-place were essentially drilled into the soft metaphyseal peri-articular bone by hand via a twisting motion. Once the needle was perceived to have passed through the cortex, the introducer was removed and a sterile 10-cc syringe was used to aspirate medullary blood. The aspirate of the ilium was performed with the needle directed into the ilium just superior to the acetabulum. The proximal femoral aspiration was performed with the needle directed towards the anterior neck of the femur, distal to the proximal femoral physis. For each of the osseous aspirations, the introducer was not removed until the needle had passed through the cortex into metaphyseal bone.

All cultures were set up to grow aerobes, anaerobes, fungi and acid fast bacteria. Cultures grown only in broth were deemed contaminants or false positives unless they accompanied a synovial fluid cell count greater than 50,000 white blood cells, were one of many similar colonies grown from two or more separate cultures, or were of a single broth colony from a known pathogen. In this series of patients, an anterior approach to the hip joint and arthrotomy were performed because of a high suspicion of septic arthritis, i.e. a purulent joint aspirate. Specimens of capsule were sent for culture and to pathology.

## Results

Despite a high suspicion of peri-articular hip infection (septic arthritis of the hip or osteomyelitis of the proximal femur or ilium) in these 36 patients, only 17 met the criteria for that diagnosis (true-positive cultures from synovial fluid, ilium or proximal femur, or capsule; or a positive culture from peripheral blood with a joint aspirate cell count greater than 50,000 WBC) (Table 1). Six of these 17 patients received antibiotics within a week preceding their presentation and treatment; 6 of the 21 patients who were culture-negative had received antibiotics within a week of the index surgical procedure. The sensitivity of hip joint synovial fluid aspirates to detect infection via positive culture (in combination with blood cultures) was only 63 %, though this increased to 100 % when the results of cultures of aspirates of the ilium and proximal femur were included. The specificities were equivalent between the two modalities: 95 % for Modality 1 vs. 90 % Modality 2) (Table 2). There were 2 of 7 patients with positive cultures from aspirates of the ilium and negative cultures from aspirates of the femur, and 7 of 12 patients with a positive culture from the femoral aspirate had a culture-negative aspirate of the ilium. There was only one patient with a positive culture of the hip joint capsule who did not also have a positive culture from aspirates of either the ilium or proximal femur (and that patient had a positive culture of their hip aspirate).

**Table 1:** Aspiration and culture results

Positive blood culture	6
Culture of joint aspirates	
True positives	9
False positives	2
Blood culture positive with joint aspirate >50,000 WBC or joint aspirate culture positive	10
Joint aspirates with >50,000 WBC	15
Culture of joint capsule	
True positives	8
False positives	1
Aspirates of ilium	
True positives	7
False positives	0
Aspirates of the proximal femur	
True positives	12
False positives	2
Aspirates of the ilium and proximal femur combined	
True positives	14
False positives	2
Meeting diagnostic criteria of peri-articular hip infection	17
Septic arthritis with peri-articular osteomyelitis	10
Septic arthritis without peri-articular osteomyelitis	4
Peri-articular osteomyelitis without septic arthritis	3

**Table 2:** Sensitivities and specificities of the aspirations to detect the infectious organisms via positive culture results

	Value (%)	95 % CI
Sensitivity, hip joint aspirates	63	36–84 %
Specificity, hip joint aspirates	95	74–100 %
Sensitivity, aspirates of the hip joint plus bone	100	77–100 %
Specificity, aspirates of the hip joint plus bone	90	67–98 %

Presumed septic arthritis of the hip was the final diagnosis for the majority of patients who were culture-negative (15/21, Table 3). Overall, this suggests that, at best with aspirations of peripheral blood, joint, ilium and femur, our ability to detect an organism in a case of true or presumed septic arthritis of the hip was 17/(17 + 15) = 53 %. This was improved from 40 % [10/(10 + 15)] using just the aspirates of blood and joint to detect infection of the eight culture-negative patients with hip aspirate cell counts greater than 50,000 WBC; five had a diagnosis of presumed septic arthritis, one juvenile idiopathic arthritis, one reactive arthritis (presumed post-streptococcal) and one peri-articular pyomyositis.

**Table 3:** Final diagnoses for 21 culture-negative patients

Diagnosis	Number
Presumed peri-articular hip infection	15
Reactive arthritis	3^a^
Juvenile idiopathic arthritis	2
Peri-articular pyomyositis	1

^a^Only one of these culture-negative patients was tested for Lyme disease and was found to be Lyme-negative

**Fig. 2:**
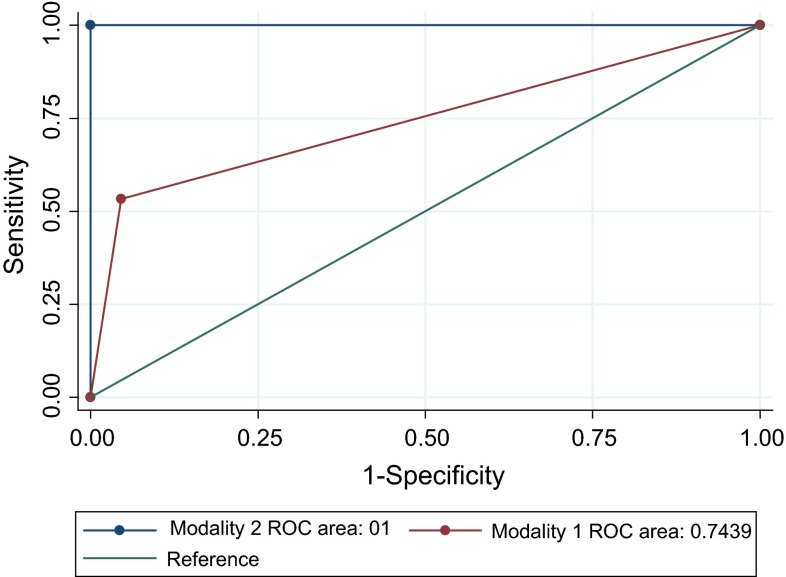
Comparison of the sensitivities of culture of joint aspirates (Modality 1) and culture of joint plus bone aspirates (Modality 2). Receiver operating characteristic (ROC) curves with area under the curve (AUC) values

We compared the sensitivities of both modalities (Modality 1: using joint aspirates alone; Modality 2: using joint aspirates plus aspirates of the ilium and femur) with ROC curves. ROC analysis showed that the AUC for Modality 2 was larger than that of Modality 1 (Mod 1 AUC = 0.744; Mod 2 AUC = 1). The Chi-square test yielded a significance probability of 0.003, suggesting that the overall performance of using positive joint aspirates plus aspirates of the ilium and femur was significantly better than using joint aspirates alone for detecting the infectious organism for a peri-articular hip infection (Fig. 2).

## Discussion

In 1986, Alderson et al. proposed that osteomyelitis and septic arthritis were two forms of the same entity. Using an animal model, they showed that bacteria injected into a joint could be found soon thereafter first on the articular cartilage and then in metaphyseal bone [23]. Likewise, injections of bacteria into metaphyseal bone led rapidly to culture-positive synovial fluid, suggesting connections between metaphyseal and epiphyseal blood vessels. Though one might explain the transmission of bacteria between metaphyseal bone and joint fluid through simple movement from metaphyseal bone into the overlying joint space for joints in which the joint capsule extends across the physis (hip, knee, ankle, elbow, shoulder), Smith hypothesised that this transmission would require a rupture of the metaphyseal cortex by the infection [1], a much slower process than that described by Alderson et al. Likely, the rate of progression of this process is somewhere between the two, given our finding of a positive culture from bone in 14 of 17 patients with positive cultures. Though this rate of approximately 80 % of cases with positive cultures from hip joint specimens having a concomitant positive culture from bone may seem high, the diagnosis of concomitant peri-articular osteomyelitis (pelvis or proximal femur) and septic arthritis of the hip is common [3, 19, 25, 26]. We have shown here that aspirating the ilium and proximal femur at the time of hip joint aspiration increases the sensitivity of the procedure to diagnose local infection, i.e. return a true-positive culture result. The advantages of identifying the infectious organism by increasing the sensitivity of the diagnostic tests (aspirations of and about the hip) are numerous. Antibiotic therapy may be focused to best treat the infection, avoiding the use of broad-spectrum antibiotics that may lead to resistant strains. Positive cultures about the hip also secure the diagnosis of a peri-articular hip infection. Whether positive cultures from aspirates of the ilium and proximal femur are harbingers of a more serious infection requiring a longer duration of treatment remains to be seen. Yet, in our experience, in the absence of any radiographic changes in the bone and after initiation of the diagnostic procedures described above, patients with positive cultures from bone when treated similarly to those diagnosed with purely a septic arthritis of the hip responded to a wash-out of the hip and antibiotic treatment in a similar fashion.

A limitation of this study is the small number of patients. Also, our overall rate of culturing an organism in cases of presumed infection at just over 50 % (17/32) is low, though consistent with our hospital rates of positive cultures for presumed septic arthritis and osteomyelitis in patients who may be immune compromised or who had previously begun antibiotic treatment. Patients with a presumed septic arthritis of the hip were not routinely screened by magnetic resonance (MR) imaging prior to aspiration in the operating room. Given the challenges to obtaining sedated and after-hours MR imaging at our institution, we reserve MR for those patients who continue to respond poorly after initial surgical treatment. Though some report advantages to pre-screening with MR prior to treatment of the presumed septic arthritis of the hip [27], we believe that this adds unnecessarily to the overall cost of treatment. In developing nations, urgent MR is rarely available or affordable; our treatment scheme of aspirating the hip as well as bone on either side of the joint is a low-cost means of obtaining informative specimens.

There are costs to processing these additional specimens, though these costs are relatively low. There are risks to the surgeon as well. The use of an 18-gauge spinal needle to penetrate cortical bone of the ilium and proximal femur may not be ideal. Placing a gauze sponge or sterile towel between the surgeon’s glove and the plastic hub of the spinal needle should prevent injury from the base of the needle, potentially exposed should the hub ride up the shaft while attempting to twist the spinal needle into bone. Alternative sampling needles such as a Jamshidi needle might be preferable, though their effectiveness has not been examined.

The aspirations of the ilium and proximal femur were performed in all of these cases without regard to the margins of the hip joint capsule; hence, intracapsular aspirations of the femur and ilium were likely routinely obtained. However, there were no known cases where a previously uninfected joint was seeded or contaminated by aspiration of an infected proximal femur or ilium, nor were there any instances where previously unaffected bone was seeded or contaminated by aspiration of bone through an infected joint. Also, following aspiration, high-dose antibiotics are routinely given intravenously, likely limiting the possibility of new infection from such contamination. It’s possible that intra-capsular aspirations of the ilium and femur in the presence of a septic hip may have led to contamination of the bone aspirates, that perhaps intra-capsular aspirations of the ilium and proximal femur serve as a means to obtain specimens from the hip joint capsule and, hence, a surrogate for a tissue culture from the capsule, as the needle likely passed through the capsule on the way to bone. Whether or not the aspirates of bone reflected bone infection or joint infection would not have impacted our treatment plan however, as we routinely treated patients with culture-positive joint and bone aspirates in the same way, as long as there were no radiographic changes seen in the bone: a wash-out of the hip for a purulent joint aspirate and intravenous antibiotics until the C-reactive protein was near normal, followed by oral antibiotics until the erythrocyte sedimentation rate was near normal.

These results support the routine aspiration of bone on either side of the hip joint at the time of hip aspiration in cases of suspected septic arthritis of the hip or peri-articular osteomyelitis. Though the population of patients analysed here included only those who also underwent arthrotomy, irrigation of the hip joint and capsular biopsy, we do not advocate for routine arthrotomy and wash-out of the hip unless the joint aspirate appears purulent. The addition of arthrotomy with wash-out of the joint and capsular biopsy should be reserved for those with a high suspicion of pus under pressure within the hip joint, such as when hip aspirates are quite cloudy or when white cell counts of aspirates are above 50,000 WBC. We included only those patients in this report who had the most informative collection of cultures, i.e. cultures from peripheral blood, hip aspirate, capsule and blood from the ilium and proximal femoral metaphyseal bone, to best determine the sensitivities and specificities of the tests to identify the likely infectious organism.

## Conclusion

Obtaining aspirates of the ilium and proximal femur at the time of hip synovial fluid aspiration increases the likelihood that the procedure will return an infectious organism. This increased likelihood of a positive culture result must be balanced against the costs of obtaining and processing the additional cultures. At a time when the virulence of infectious organisms may vary widely, having positive culture results from a patient with a septic hip or peri-articular hip infection may most efficiently and effectively guide antibiotic treatment. Adding a routine aspiration of the ilium and proximal femur substantially increases the likelihood that aspirates obtained will return a positive culture in the setting of a presumed septic arthritis of the hip.

## References

[1] Smith T (1874). On the acute arthritis of infants. St Bartholomew’s Hosp Rep.

[2] Morrey BF, Bianco AJ, Rhodes KH (1976). Suppurative arthritis of the hip in children. J Bone Joint Surg Am.

[3] Caksen H, Oztürk MK, Uzüm K, Yüksel S, Ustünbaş HB, Per H (2000). Septic arthritis in childhood. Pediatr Int.

[4] Badgley CE, Yglesias L, Perham WS, Snyder CH (1936). Study of the end results in 113 cases of septic hips. J Bone Joint Surg.

[5] Heberling JA (1941). A review of two hundred and one cases of suppurative arthritis. J Bone Joint Surg.

[6] Samilson RL, Bersani FA, Watkins MB (1958). Acute suppurative arthritis in infants and children; the importance of early diagnosis and surgical drainage. Pediatrics.

[7] Griffin PP (1967). Bone and joint infections in children. Pediatr Clin North Am.

[8] Stetson JW, DePonte RJ, Southwick WO (1968). Acute septic arthritis of the hip in children. Clin Orthop Relat Res.

[9] Paterson DC (1970). Acute suppurative arthritis in infancy and childhood. J Bone Joint Surg Br.

[10] Gillespie R (1973). Septic arthritis of childhood. Clin Orthop Relat Res.

[11] Lunseth PA, Heiple KG (1979). Prognosis in septic arthritis of the hip in children. Clin Orthop Relat Res.

[12] Jackson MA, Nelson JD (1982). Etiology and medical management of acute suppurative bone and joint infections in pediatric patients. J Pediatr Orthop.

[13] Fabry G, Meire E (1983). Septic arthritis of the hip in children: poor results after late and inadequate treatment. J Pediatr Orthop.

[14] Wilson NI, Di Paola M (1986). Acute septic arthritis in infancy and childhood. 10 years’ experience. J Bone Joint Surg Br.

[15] Chen CH, Lee ZL, Yang WE, Lin TY, Shih CH (1993). Acute septic arthritis of the hip in children—clinical analyses of 31 cases. Changgeng Yi Xue Za Zhi.

[16] Vidigal EC, Vidigal EC, Fernandes JL (1997). Vidigal EC, Fernandes JL. Avascular necrosis as a complication of septic arthritis of the hip in children. Int Orthop.

[17] Marx RG, Wright JG (1999). Slipped capital femoral epiphysis after septic arthritis of the hip in an adolescent: report of a case. Can J Surg.

[18] Heyworth BE, Shore BJ, Donohue KS, Miller PE, Kocher MS, Glotzbecker MP (2015). Management of pediatric patients with synovial fluid white blood-cell counts of 25,000 to 75,000 cells/mm^3^ after aspiration of the hip. J Bone Joint Surg Am.

[19] Chen CE, Ko JY, Li CC, Wang CJ (2001). Acute septic arthritis of the hip in children. Arch Orthop Trauma Surg.

[20] Howard-Jones AR, Isaacs D, Gibbons PJ (2013). Twelve-month outcome following septic arthritis in children. J Pediatr Orthop B.

[21] Kocher MS, Mandiga R, Murphy JM, Goldmann D, Harper M, Sundel R (2003). A clinical practice guideline for treatment of septic arthritis in children: efficacy in improving process of care and effect on outcome of septic arthritis of the hip. J Bone Joint Surg Am.

[22] Caird MS, Flynn JM, Leung YL, Millman JE, D’Italia JG, Dormans JP (2006). Factors distinguishing septic arthritis from transient synovitis of the hip in children. A prospective study. J Bone Joint Surg Am.

[23] Alderson M, Speers D, Emslie K, Nade S (1986). Acute haematogenous osteomyelitis and septic arthritis—a single disease. An hypothesis based upon the presence of transphyseal blood vessels. J Bone Joint Surg Br.

[24] Kocher MS, Zurakowski D, Kasser JR (1999). Differentiating between septic arthritis and transient synovitis of the hip in children: an evidence-based clinical prediction algorithm. J Bone Joint Surg Am.

[25] Ogden JA (1979). Pediatric osteomyelitis and septic arthritis: the pathology of neonatal disease. Yale J Biol Med.

[26] Bennett OM, Namnyak SS (1992). Acute septic arthritis of the hip joint in infancy and childhood. Clin Orthop Relat Res.

[27] Mazur JM, Ross G, Cummings J, Hahn GA, McCluskey WP (1995). Usefulness of magnetic resonance imaging for the diagnosis of acute musculoskeletal infections in children. J Pediatr Orthop.

